# The Role of FcRn in Antigen Presentation

**DOI:** 10.3389/fimmu.2014.00408

**Published:** 2014-08-27

**Authors:** Kristi Baker, Timo Rath, Michal Pyzik, Richard S. Blumberg

**Affiliations:** ^1^Division of Gastroenterology, Department of Medicine, Brigham and Women’s Hospital, Harvard Medical School, Boston, MA, USA; ^2^Division of Gastroenterology, Department of Medicine, Erlangen University Hospital, Friedrich Alexander University Erlangen-Nueremberg, Erlangen, Germany; ^3^Harvard Digestive Diseases Center, Boston, MA, USA

**Keywords:** FcRn, IgG, antigen presentation, dendritic cells, immune complex

## Abstract

Immunoglobulins are unique molecules capable of simultaneously recognizing a diverse array of antigens and themselves being recognized by a broad array of receptors. The abundance specifically of the IgG subclass and the variety of signaling receptors to which it binds render this an important immunomodulatory molecule. In addition to the classical Fcγ receptors that bind IgG at the cell surface, the neonatal Fc receptor (FcRn) is a lifelong resident of the endolysosomal system of most hematopoietic cells where it determines the intracellular fate of both IgG and IgG-containing immune complexes (IgG IC). Cross-linking of FcRn by multivalent IgG IC within antigen presenting cells such as dendritic cells initiates specific mechanisms that result in trafficking of the antigen-bearing IgG IC into compartments from which the antigen can successfully be processed into peptide epitopes compatible with loading onto both major histocompatibility complex class I and II molecules. In turn, this enables the synchronous activation of both CD4^+^ and CD8^+^ T cell responses against the cognate antigen, thereby bridging the gap between the humoral and cellular branches of the adaptive immune response. Critically, FcRn-driven T cell priming is efficient at very low doses of antigen due to the exquisite sensitivity of the IgG-mediated antigen delivery system through which it operates. FcRn-mediated antigen presentation has important consequences in tissue compartments replete with IgG and serves not only to determine homeostatic immune activation at a variety of sites but also to induce inflammatory responses upon exposure to antigens perceived as foreign. Therapeutically targeting the pathway by which FcRn enables T cell activation in response to IgG IC is thus a highly attractive prospect not only for the treatment of diseases that are driven by immune complexes but also for manipulating local immune responses against defined antigens such as those present during infections and cancer.

## Introduction

Coordination of immune responses is of key importance to the maintenance of homeostasis within multicellular organisms. Fundamental to this process is the recognition, processing, and presentation of antigenic agents that allows integration of the various branches of the innate and adaptive immune systems that cooperate to confer maximal protective immunity. Antigen detection, which serves as the initiating event in this cascade, occurs via numerous mechanisms having varying levels of sensitivity and specificity. How an antigen is detected depends at once on the nature of the antigen itself as well as on the particular immune cell that detects it. Whereas small soluble antigens can be taken up passively via macropinocytosis or fluid phase endocytosis, larger antigens require processes such as phagocytosis for cellular entry. Specificity for antigen detection, uptake, and/or processing is conferred by cellular receptors that may bind to a unique ligand, such as insulin-like growth factor receptor 1 (IGFR1), or to a conserved motif present on many ligands, such as the mannose receptor (MR) DC-SIGN. In each case, ligand binding by the receptor can not only initiate ligand internalization but also trigger additional signaling cascades, which exert direct or indirect effects on subsequent antigen presentation.

Recognition of IgG by Fcγ receptors (FcγRs) represents an important strategy that enables the delivery of unique antigenic determinants in the form of an IgG immune complex (IgG IC) via binding of the conserved Fc receptor (FcRn) domains on IgG to their receptor. In antigen presenting cells (APCs), this process is initiated at the cell surface by FcγR ligation, which triggers both the internalization of IgG and its delivery into endocytic vesicles. Importantly, while monomeric IgG is efficiently recycled from these compartments to the cell surface, antigen-containing IgG immune complexes (IgG IC) are instead trafficked into vesicles where processing of the complexed antigen releases epitopes that are loaded onto MHC class I (MHC-I) and class II (MHC-II) molecules that subsequently stimulate the activation of cognate CD8^+^ and CD4^+^ T cells. Importantly, following entry into the cell, routing of the IgG or IgG IC through the maze of acidic endocytic compartments is mediated neither by FcγR nor by non-specific distributive mechanisms but rather by the specific binding of IgG to its intracellular receptor, the neonatal Fc receptor (FcRn).

Best known for its roles in protecting circulating IgG from catabolism and mediating IgG transcytosis across polarized epithelial cells at mucosal surfaces, FcRn also plays a critical role in the immune system. Lifelong, high level expression of FcRn in APC such as dendritic cells (DC), macrophages, and B cells enables the specific intracellular trafficking of IgG and antigen-containing IgG IC through the endolysosomal system. Importantly, in the case of the latter, this mechanism results in the delivery of complexed antigen into compartments in which the local degradative conditions favor epitope conservation and the loading of immunostimulatory antigenic epitopes onto MHC-I and MHC-II. FcRn expression within APC thus has important immunological consequences for the generation of targeted T cell mediated immunity following the triggering of a generic cellular entry pathway by the conserved Fc portion of IgG. Rather uniquely, FcRn-mediated activation of T cells in response to IgG IC serves to integrate the humoral and cell-mediated branches of the adaptive immune system and to coordinate the innate immune system, thereby promoting maximal protective immunity.

## Antigen Presentation

### MHC class I and II peptide loading

Functionally, antigen presentation has evolved as a mechanism by which T cells can monitor the antigenic composition of the body for the presence of potential pathogens. Given the immense variability in antigen structure and composition, the development of a systematized way for T cells to rapidly and efficiently monitor antigenic diversity in their environment can be considered essential for maximizing organism survival. The major histocompatibility complex (MHC) molecules, which are expressed at the cell surface in complex with antigenic epitopes, present the T cell with a conserved recognition structure, the MHC molecule itself, in conjunction with a unique peptide that transmits information about the antigenic milieu. Collectively, T cells are thus able to monitor an immense array of antigens due to a multitude of clonotypically expressed T cell receptors (TCR).

The peptide binding characteristics differ among different classes of MHC molecules. MHC-I molecules, which are recognized by CD8^+^ T cells, bind peptides of 8–11 amino acids in length that are generated largely via the process of proteasomal degradation in the cytosol (1). Peptide loading onto MHC-I occurs in the endoplasmic reticulum (ER) after cytoplasmic proteasomal digestion and subsequent peptide import by the transporter associated with antigen processing (TAP) across the ER membrane. The vast majority of peptides presented by MHC-I are thus endogenous to the cell itself (1, 2). In specialized cell types, particularly DC, exogenously acquired antigens can be presented by MHC-I through a process known as cross-presentation as discussed further below (3). MHC-II molecules, which educate or activate CD4^+^ T cells in the thymus or periphery, respectively, bind peptides of 10–30 amino acids in length, which are typically exogenously derived and processed by lysosomal proteolysis. While most cells engage in MHC-I presentation, APCs are proficient at both MHC-I and MHC-II presentation due to the specialized intracellular machinery that they possess.

### Processing Pathways for Exogenous Antigens

The generation of peptides suitable for loading onto MHC molecules is dictated at once by the nature of the antigen, the route of uptake, and additional signals to which the cell is exposed. With respect to the contribution of the antigen itself, certain epitopes of a protein often exhibit immunodominance leading to the preferential processing and presentation of such epitopes over others (4, 5). Many factors contribute to the preferential selection of certain epitopes, including the activation state of the APC, the relative protein abundance, the availability and activity of enzymes responsible for antigen processing, and previous antigen exposure. Importantly, immunodominance can have profound implications not only for shaping the immediate direction of an immune response but also the nature of future immune response by altering memory T cell pools (1, 4).

Processing of exogenous antigen for loading onto MHC-II molecules is initiated upon entry into the early endosome. As the antigen moves progressively deeper into the endolysosomal system, the pH of the vesicles progressively decreases from the mildly acidic environment of the early endosome to the highly acidic milieu of the lysosome (6). Degradation of the antigen occurs largely by the actions of various enzymes, particularly cathepsin proteases and the γ-interferon-inducible lysosomal thiolreductase (GILT), whose activity is regulated by the local pH (7). While there is considerable heterogeneity in the morphology and content of the various endolysosomal compartments and MHC-II molecules can be found throughout compartments at various stages of maturation, the majority of evidence indicates that epitope loading onto MHC-II occurs in the late endosome, which has also been referred to as an MHC-II compartment, where numerous chaperones facilitate this process (8). However, conditions within both the late endosome and phagosome are thought to be optimal for the generation of peptides ideal for MHC-II loading and trafficking of such peptides between these two compartments is known to occur (1, 2). Given the dependence of antigenic processing on pH, the efficiency of productive antigen processing varies across cell types with different endolysosomal pH ranges (6, 9). The highly acidic environment within macrophages enables the efficient generation of peptides suitable for loading on MHC-II but results in the loss of many potentially antigenic epitopes due to the harsh processing conditions. In contrast, the endosomal pH is much more strictly buffered within DC and generally remains in a much more neutral range due to the alkalinizing actions of the NADPH oxidase NOX2, which is recruited to phagosomal and endosomal membranes (10). Thus, a different set of MHC-II-compatible peptides emerges from antigen processing in different cell types.

Cross-presentation, the loading of exogenously derived antigens onto MHC-I, is highly cell type-specific and has so far only been documented to occur efficiently in DC. Given that epitopes suitable for loading onto MHC-I are more susceptible to degradation than MHC-II-compatible epitopes, the milder processing conditions within DC are thought to provide the ideal environment for their generation (10–12). Subsequent to cell uptake by endocytosis or phagocytosis, exogenous antigens within DC are exposed to a near neutral endosomal/phagosomal pH resulting from an incomplete assembly of the vacuolar (V)-ATPase on vesicle membranes as well as the Rac2 and Rab27a-dependent recruitment of NOX2 (10, 11, 13). The generation of reactive oxygen species (ROS) by NOX2 and subsequent consumption of protons in the compartment results in a near neutral pH. Additionally, proteasomal recruitment of lysosomal proteases has been shown to be lower in DC compared to macrophages (14). Such mild conditions alone, however, cannot produce peptides suitable for MHC-I loading and a critical step in cross-presentation is the retro-translocation of antigen out of the endosomal compartment and into the cytosol where it is processed by the proteasome in a manner akin to endogenous proteins (15). The precise mechanism for this retro-translocation is not known but the process of ER-associated degradation (ERAD) and, specifically, the actions of the Sec61 retrotranslocon have been implicated in the process (15, 16). The peptides generated by proteasomal processing are then imported into the ER, where the majority of MHC-I is loaded with peptide via the actions of TAP (15). Further peptide trimming within the ER is mediated by ER aminopeptidases (ERAPs) before nascent MHC-I molecules are loaded with the final epitope assisted by chaperones such as calnexin and calreticulin (17). While the involvement of the proteasome and ER machinery in cross-presentation is well accepted, it has been proposed that the phagosome or endosome itself may form a self-sufficient cross-presentation compartment that contains significant amounts of ER-associated proteins (18, 19). These include Sec61, TAP, IRAP (an N-terminal peptidase very similar to ERAPs), and MHC-I itself (18–20). It is perhaps most likely that these pathways are not mutually exclusive and that the specific route through which a given antigen is processed depends upon numerous factors.

This raises the important principle that for both MHC-I and MHC-II antigen processing, there exists a very tight link between the generation of suitable epitopes for MHC loading and the environment within the antigen processing compartments. Thus, regulation of antigen processing is highly susceptible to cellular and antigen context. Exposure of APC to stimuli such as TLR ligands, pro- or anti-inflammatory cytokines, or hormones can exert significant impact upon the phagolysosomal processing efficiency by varying the pH, protease expression, endosomal maturation processes, or signaling cascades initiated upon antigen internalization (21–25).

## Receptor-Mediated Antigen Uptake

The means by which an antigen is internalized is known to significantly impact its intracellular handling by the cell. Many receptors, particularly PRR, have been identified that promote antigen presentation either by increasing the rate of antigen uptake by other cell surface receptors or by initiating signaling pathways, which influence antigen handling once they are internalized. Importantly, different receptors can exert different effects on MHC-II and MHC-I antigen presentation and this has been linked to the routing of antigen to different compartments by the various receptors. Thus, whereas internalization of an antigen by scavenger receptors enhances MHC-II loading and the activation of CD4^+^ T cells, antigen uptake via the MR promotes MHC-I loading and CD8^+^ T cell activation, likely by enhancing antigen export to the cytosol (26–28). In contrast, C-type lectin receptors such as CLEC9, Dectin-1, and DEC-205 (lymphocyte antigen 75), which are expressed on macrophages and DC, promotes antigen presentation via both MHC-I and MHC-II (29–33). Indeed, many studies have attempted to target antigen to these various receptors in order to stimulate directed antigen presentation and generate enhanced antigen-targeted immune responses. The strategy most commonly employed in these experiments has been to either conjugate antigen or to fuse antigen with a receptor-specific monoclonal antibody (32, 34, 35). Thus, while each of these strategies has been shown to effectively enhance antigen-specific immune responses, it cannot be excluded that such responses were partially mediated by binding of the Fc portion of the targeting antibody to FcγR, which would also enhance antigen presentation (36–38).

### Classical Fcγ Receptors

Cell surface expression of IgG binding receptors is a common feature of APC that allows them to react with high sensitivity to both monomeric IgG and antigen-containing IgG IC. The family of classical FcγR consists of several members with differential binding affinities and signaling capabilities, which transmit a diverse range of responses upon ligation and thereby form a highly tunable system for the regulation of immune responses.

Broadly, FcγR can be separated into activating and inhibiting receptors, all of which likely arose from a series of gene deletion/duplication and inter-gene recombination events (39). In humans, activating receptors include hFcγRI, hFcγRIIA, hFcγRIIC, hFcγRIIIA, and hFcγRIIIB, whereas in mice these include mFcγRI, mFcγRIII, and mFcγRIV (37, 38). Activation by these receptors is mediated by an immunoreceptor tyrosine-based activation motif (ITAM) that is located either directly in the cytoplasmic tail of the receptor, in the case of FcγRII homologs, or in the tail of the Fc receptor common γ-chain (FCER1G), which associates tightly with the cytoplasmic tails of FcγRI, hFcγRIIIA, and mFcγRIV. Ligand binding induces phosphorylation of the ITAM motifs by proto-oncogene tyrosine-protein kinase Src (SRC) family kinases, which, in turn, trigger the recruitment and docking of spleen tyrosine kinase (SYK) via its two SRC homology 2 (SH2) domains. Subsequent autophosphorylation of SYK initiates a complex web of intracellular signaling pathways including activation of the mitogen-activated protein kinase (MAPK), phosphoinositide 3-kinase (PI-3K), and protein kinase C (PKC) cascades (40–42). Inhibitory FcγRs include FcγRIIB orthologs in both mouse and human systems, which possess an immunoreceptor tyrosine-based inhibitory motif (ITIM) in their cytoplasmic tails. Upon ligand binding, ITIM phosphorylation induces the recruitment of phosphatases such as SHIP-1 [inositol polyphosphate-5-phosphatase (INPP5D)], which drive subsequent immunosuppression (37, 43). Numerous polymorphisms, splice variants, and copy number variations for FcγR family members have been documented in both humans and mice, many of which were shown to alter either ligand binding, downstream signaling or be associated as risk factors for human disease (44–46).

Importantly, the range of ligand binding affinities varies widely across FcγR family members. In humans, FcγRI has the highest affinity for monomeric IgG1, the lowest for monomeric IgG2, and intermediate affinity for IgG3 and IgG4. Given this high affinity for monomeric IgG, it is widely believed that most FcγRI is saturated at steady state in the presence of physiological serum IgG concentrations (47). FcγRII and FcγRIII variants exhibit relatively poor binding for monomeric IgG but a much higher binding for multimeric IgG, such as is found in IgG IC, due to the ability of such multivalent ligands to crosslink surface receptors. Furthermore, it is increasingly being appreciated that expression of FcγR varies widely across types of APC. Whereas transcript analysis has revealed that monocytes, macrophages, and monocyte-derived DC (moDC) from mice express relatively high levels of all FcγR, expression in DC subsets is considerably lower (38). Both XCR1^+^ and CD172α^+^ conventional DC (cDC) as well as plasmacytoid DC (pDC) predominantly express FcγRIIA and FcγRIIB transcripts with lower levels of expression of other FcγR. Indeed, three groups have recently identified expression of FcγRI as a highly sensitive marker for the differentiation of moDC from cDC (48–52). These differences in affinity for IgG of different subclasses and valencies as well as differential distribution of receptor expression across APC types have important implications for the fine tuning of immune regulation, as will be discussed below.

Internalization by FcγR has clearly been shown to increase the ability of APC to present antigen contained within an IgG IC and thus to stimulate CD4^+^ and CD8^+^ T cell responses (36, 53–57). FcγR ligation by IgG leads to uptake of bound ligands by either clathrin-dependent receptor-mediated endocytosis, if the ligand is a small IgG IC, or by actin- and PI-3K-dependent phagocytosis, if the ligand is a large IgG IC (58). FcγR-mediated internalization, which is dependent on the common γ-chain, directs IgG IC into an intracellular pathway that is conducive to antigen presentation while simultaneously inducing maturation of the internalizing DC (56, 59). Importantly, however, FcγR binding to IgG occurs at neutral pH such as is found at the cell surface, but not in the acidic pH range that is found in the endolysosomal system (60). FcγRs are thus unable to bind their cargo intracellularly and such that they release it in the acidifying endosomes soon after internalization. Given the inability of FcγR to directly route IgG IC through specific antigen processing compartments, it was thus unclear for an extended period of time whether the contribution of FcγR to enhanced antigen presentation was simply the result of increased antigen uptake, of signaling pathways initiated by IgG IC-mediated FcγR cross-linking or by an unidentified mechanism.

## FcRn: An Intracellular IgG Binding Receptor

The discovery of an intracellular IgG binding receptor distributed throughout the endolysosomal system within APC and which exhibits specific binding only under acidic conditions presented the possibility that IgG IC could be specifically trafficked toward antigen processing compartments subsequent to FcγR-mediated uptake.

Fc receptor was first identified in the intestinal epithelial cells of neonatal rodents and thus came to be known as the neonatal Fc receptor, FcRn (61). Indeed, FcRn remains best known for the critical role it plays in transcytosis of IgG across intestinal epithelial cells at mucosal barriers where it serves the important function of delivering maternal IgG to neonatal rodents with undeveloped immune systems (62–64). In the case of humans, such passive acquisition of IgG in an FcRn-dependent manner occurs antenatally via the placenta (65, 66). However, discovery of FcRn in the hepatocytes of adult rats was indicative that FcRn function was not limited to the neonatal system and this was later substantiated by the documentation of FcRn expression in adult human intestinal epithelial cells (64, 67). Indeed, the other well-known role for FcRn, protecting its serum ligands (IgG and albumin) from catabolism via recycling, is an important process ongoing throughout life (68, 69). Lifelong FcRn expression has now been documented in a wide range of parenchymal cells in various tissues including intestinal epithelial cells, airway epithelial cells, placental syncytiotrophoblasts, hepatocytes, and endothelial cells in species ranging from humans to rodents to camels (66, 67, 70–72). Critically, discovery of high level FcRn expression in hematopoietic cells such as B cells, macrophages, and DC raised the possibility that FcRn could be directly involved in the presentation of IgG-complexed antigens to T cells, which themselves do not express FcRn (73, 74).

**Figure 1:**
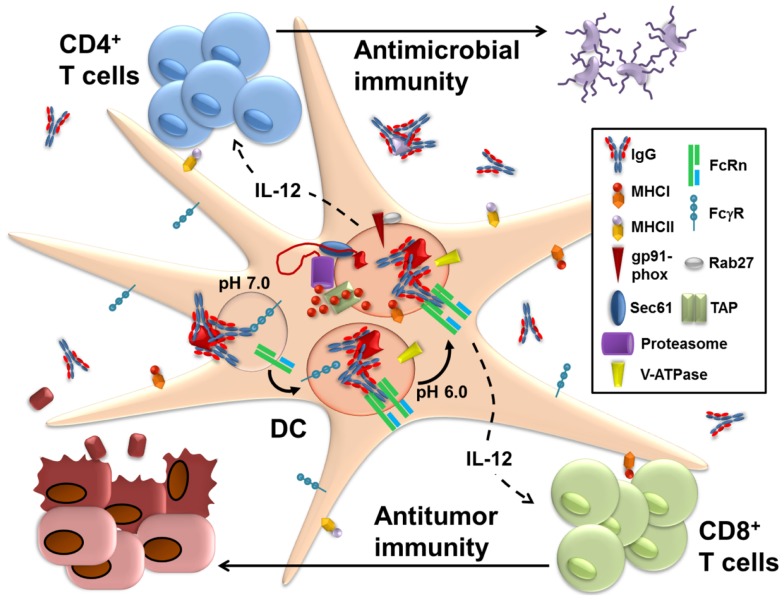
**FcRn within dendritic cells enables the presentation of IgG-complexed antigens to CD4^+^ T cells and CD8^+^ T cells**. Following IgG opsonization of antigens from sources such as microbes or tumors, the immune complexes (IgG IC) bind to FcγR on the surface of dendritic cells (DC). This initiates receptor-mediated endocytosis, which delivers the IgG into the endolysosomal system of compartments. As these vesicles become more acidic during their maturation due to the recruitment of the vacuolar ATPase (V-ATPase), IgG IC dissociate from FcγR and bind instead to FcRn, which enables the subsequent trafficking of the IgG IC into antigen processing pathways that promote the generation of epitopes compatible with loading onto MHC class I and MHC class II. Machinery known to be involved in the presentation of soluble antigens for MHC class I processing and presentation, such as gp91-phox, Rab27, Sec61, TAP (transporter associated with antigen processing), and the proteasome, are all preferentially recruited to the IgG IC-containing intracellular vesicles upon ligation of FcRn. Less information is available on the compartments involved in FcRn-facilitated MHC class II restricted presentation and it remains unknown whether this occurs within the same intracellular compartment as MHC class I-restricted processing. Once at the surface, these peptide loaded MHC molecules are then able to prime CD8^+^ and CD4^+^ T cells, respectively. Furthermore, ligation of FcRn by IgG IC induces the production of IL-12 by the DC. The secreted IL-12 acts upon the primed CD4^+^ T cells to induce Th1 polarization and upon the CD8^+^ T cells to promote activation and cytotoxicity. FcRn within DC thus contributes to the activation of cell-mediated adaptive immune responses that contribute to pathogen eradication and tumor protection.

Several key features of FcRn biology render it an ideal candidate molecule for participation in IgG IC antigen presentation (Figure 1). FcRn, which is encoded by the *Fcgrt* gene, is an MHC-I related molecule that associates directly with the β2-microglobulin chain that enables its proper assembly and stability (75). FcRn is located primarily intracellularly within the endolysosomal system of APC and less so on the cell surface where it could theoretically compete for IgG binding with FcγR (76). The critical binding site for FcRn on the IgG Fc region involves the I253, H310, and H435 residues within the CH2:CH3 domains of IgG Fc and is distinct from where FcγR binding to IgG occurs (77–84). In contrast to FcγRs, binding is entirely independent of IgG glycosylation. Thus, the simultaneous ligation of an IgG molecule by both classes of receptor would be possible. FcRn binds IgG with very high affinity of approximately 10 mM in a 2:1 stoichiometric ratio such that binding of multimeric IgG IC would be expected to result in significant cross-linking of FcRn (85–87). Perhaps most importantly for the purposes of antigen presentation, binding of FcRn to IgG occurs in the acidic pH range of 4.5–6.5 due to protonation of the two critical histidine residues under low pH conditions and the stabilization of the resulting salt bridges with acidic residues in FcRn by a neighboring isoleucine residue (88, 89). These pHs correspond to the pH range that is found across various compartments of the endolysosomal system, thereby enabling direct high affinity binding of FcRn to its ligand throughout the compartments where the processing for antigen presentation is known to occur (6, 90). Furthermore, FcRn is known to bind and actively traffic monomeric IgG across polarized epithelial cells and within endothelial cells, thereby establishing a direct precedent for its ability to engage in endosomal IgG routing and consistent with a dileucine motif in its cytoplasmic tail (91–94). FcRn thus possesses ideal characteristics enabling it to function downstream of cell surface FcγR in facilitating the processing of IgG-complexed antigens for presentation on MHC molecules.

### FcRn in MHC-II Antigen Presentation

The first indication that FcRn might enhance antigen presentation on MHC-II came from a study that examined the transfer of IgG-complexed ovalbumin (OVA) across the intestinal barrier and the delivery of IgG IC to local DC in the lamina propria and mesenteric lymph nodes (mLN) (95). Subsequent to oral administration of fluorescently labeled IgG IC, fluorescent signal was detected in cells of the intestinal lamina propria and CD11c^+^ cells of the mLN in mice expressing a human FcRn transgene (*hFCGRT/hB2M/mFcgrt*^−/−^) but not in mice deficient in expression of FcRn (*Fcgrt*^−/−^). This result clearly indicated that efficient uptake of IgG IC by mucosal DC occurs preferentially in FcRn-expressing mice and is consistent with the demonstration that FcRn on neutrophils increases the rate of phagocytosis of IgG IC (96). Coculture of OVA-specific CD4^+^ T cells with CD11c^+^ cells isolated from the mLN of mice fed OVA IgG IC induced T cell activation only if the mice expressed FcRn. These data strongly indicated that DC were capable of activating T cells in response to IgG IC but did not conclusively show that FcRn’s locus of action was in the DC since drastically less IgG IC would reach DC in *Fcgrt*^−/−^ animals given the inability of FcRn-deficient epithelium to transcytose lumenal IgG IC. Similarly, the demonstration that FcRn contributes to protection from the intestinal pathogen *Citrobacter rodentium* in the presence of specific IgG and that proliferation of *C. rodentium*-specific CD4^+^ T cells in the mLN was only observed in mice expressing an epithelial-specific FcRn transgene cannot be said to indicate a direct role for FcRn in antigen presentation (97). Thus, while these studies elegantly demonstrated the role of FcRn in delivering lumenal IgG IC to the mucosal immune system and thereby enabling monitoring of its contents, they do not conclusively demonstrate a role for FcRn in the direct presentation of antigen by APC.

Conclusive evidence supporting a direct role for FcRn in mediating MHC-II antigen presentation was achieved in a study that was the first to document that FcRn responds differentially to monomeric IgG and multimeric IgG IC. Whereas monomeric IgG is protected in the circulation by FcRn in both hematopoietic and parenchymal cells, leading to profound hypogammaglobulinemia in *Fcgrt*^−/−^ animals, multimeric IgG IC are much more rapidly cleared from the circulation of wild type (WT) mice than monomeric IgG (69, 76, 98). Importantly, bone marrow chimeras in which WT mice were reconstituted with bone marrow cells from either WT or *Fcgrt*^−/−^ animals clearly indicated that hematopoietic cells were responsible for the rapid loss of IgG IC from circulation. This finding suggested that IgG IC were being degraded within hematopoietic cells, a process that might lead to the generation of epitopes for loading onto MHC molecules. Indeed, DC isolated from WT mice were able to efficiently prime CD4^+^ T cells when they had been pre-incubated with IgG IC formed in the presence of as little as 0.05 μg/ml of OVA antigen. DC isolated from *Fcgrt*^−/−^ animals, in contrast, required antigen concentrations nearly 1000-fold higher in order to induce equivalent CD4^+^ T cell activation, indicating that FcRn greatly enhances the ability of DC to generate MHC-II compatible epitopes from IgG IC-delivered antigens. These results were confirmed with the use of IHH-IgG, a chimeric antibody molecule engineered to have alanine substitutions in three Fc-domain residues critical for binding to FcRn (I253A, H310A, and H435A) and thereby unable to bind to FcRn while retaining FcγR binding affinity (99). WT DC incubated with IHH-IgG IC failed to induce efficient CD4^+^ T cell proliferation compared to those incubated with the non-mutated parental IgG. Confocal microscopy on human moDC loaded with fluorescently labeled IgG IC revealed strong colocalization with FcRn within 5 min of IgG IC exposure. Furthermore, whereas IgG IC were directed into a LAMP1^+^ compartment within 30 min, IHH-IgG IC did not co-localize with LAMP1 and instead seemed to disappear from the cell entirely in this time frame. Given that LAMP1 is a well-known lysosomal marker, these data indicate that FcRn functions to direct IgG IC into lysosomes, which is a compartment in which MHC-II compatible epitopes are known to be efficiently generated (1). Together, these findings firmly establish that FcRn in DC contributes directly to activation of CD4^+^ T cells via a mechanism involving degradation of IgG-complexed antigen in a manner, which generates epitopes compatible with MHC-II loading.

Details of the mechanism by which FcRn promotes MHC-II antigen presentation have not yet been fully elucidated; however, several studies have identified important components of the process. Whereas FcRn enhanced antigen presentation in both bone marrow-derived macrophages (BMMC) and dendritic cells (BMDC) following FcRn-mediated endocytosis, this effect was lost in BMDC, but not BMMC, when the antigen was taken up via phagocytosis (100). This was shown to be due to the failure of phagocytosing BMDC to adequately acidify their phagosomes subsequent to IgG IC internalization, which is a known characteristic of DC but is non-permissive for FcRn–IgG binding (1, 101). In contrast, BMMC efficiently acidified both phagosomes and endosomes thereby promoting binding of FcRn to its IgG IC ligand. Sufficient acidification of the endocytic environment is thus a key factor for FcRn-mediated MHC-II antigen presentation. Interestingly, FcRn has been found to interact directly with the invariant chain (CD74) that is critical for assembly and trafficking of MHC-II molecules and is upregulated by inflammatory stimuli (1, 102). In fact, trafficking of FcRn into the late endosome and lysosome of epithelial cells, macrophages, and DC was shown to be entirely dependent upon its co-expression with the invariant chain. In contrast, the cytoplasmic tail of FcRn itself played almost no role in determining FcRn localization throughout these compartments since reconstitution of cells with a tailless FcRn mutant had little impact on its endolysosomal distribution as long as the invariant chain was also present in the cells. The relevant residues for mediating FcRn trafficking were found to be two dileucine-based motifs located in the endosomal sorting signal cytoplasmic tail of the invariant chain (103). The interaction between FcRn and the invariant chain was initiated in the ER and persisted throughout the endosomal system. It thus appears that FcRn shares a very similar intracellular sorting route to MHC-II whose endolysosomal distribution has also been shown to depend on its association with the invariant chain (104, 105). This is particularly interesting, given the recent findings that phagosomes behave autonomously in terms of cargo degradation and MHC-II antigen presentation and that the resulting CD4^+^ T cell activation was significantly accelerated when antigen was delivered in the form of an IgG IC (106). Thus, the tight overlap between the trafficking of the two molecules places FcRn in an ideal position to deliver IgG-complexed antigens for loading onto proximal MHC-II molecules subsequent to their processing by lysosomal proteases.

### FcRn in MHC-I Antigen Cross-Presentation

Conclusive evidence that FcRn in DC contributes to CD4^+^ T cell activation in response to IgG-complexed antigens raised the likelihood that it might also mechanistically contribute to the cross-presentation of antigens in IgG IC. Numerous studies demonstrating that IgG IC efficiently deliver antigens into a cross-presentation pathway strongly supported this hypothesis (56, 59, 107). Importantly, each of these studies identified FcγR as being critical for initiating cross-presentation and, while endosome-to-cytosol transport (59), proteasomal processing, and TAP1–TAP2 transport (56) were identified as important for FcγR-dependent antigen processing, the intracellular routing of the IgG IC itself was not investigated.

In contrast to MHC-II antigen presentation, which is carried out by most types of APC, cross-presentation occurs selectively within DC. Specifically, the majority of literature on cross-presentation has identified the CD8^+^ DC subset in mice, or their human BDCA-3^+^ DC counterparts, as being by far the most competent cross-presenters compared to even other DC subsets (108–110). Mechanistically, this has been explained by their non-monocytic lineage, which confers a distinctly neutral endosomal pH enabling preservation of antigenic integrity for the generation of intact epitopes conducive to MHC-I loading (6, 10, 11, 101). A caveat to these findings on the competency of DC subsets for cross-presentation is that most studies have examined the process of cross-presentation as it pertains to soluble protein antigen. The one study having examined cross-presentation of IgG IC by different DC subsets reported that CD8^−^ DC were capable of cross-presenting IgG IC at a similar magnitude to their CD8^+^ counterparts but that there was an absolute requirement for expression of functional FcγR only on the CD8^−^ DC (107).

Initial studies of the ability of FcRn to mediate cross-presentation of IgG-complexed antigen were carried out in CD8^+^CD11b^−^ and CD8^−^CD11b^+^ DC over a wide range of antigen concentrations. Whereas cross-presentation of antigen delivered as an IgG IC was not affected by FcRn in CD8^+^CD11b^−^ DC, cross-presentation of IgG-complexed antigens by CD8^−^CD11b^+^ DC was significantly impaired in the absence of FcRn (60). CD8^−^CD11b^+^ DC expressing FcRn were able to activate a robust CD8^+^ T cell response when exposed to antigen concentrations 100-fold lower than DC from *Fcgrt*^−/−^ mice. Similar results were seen when CD8^−^CD11b^+^ DC were exposed to IHH-IgG IC, which were no more effective at priming CD8^+^ T cells than soluble antigen. Importantly, whereas CD8^+^CD11b^−^ DC were found to activate CD8^+^ T cells in response to the cross-presentation of 5–10 μg/ml soluble antigen, as reported in the literature, FcRn enabled CD8^−^CD11b^+^ DC to stimulate similar magnitudes of CD8^+^ T cell activation in response to as little as 0.5 μg/ml of IgG-complexed antigen (10, 11, 60, 101, 111). In fact, at high antigen concentrations or following prolonged incubation times where soluble uptake of antigen is efficient, FcRn was not observed to promote cross-presentation (100). Consistent with initial studies showing the importance of FcγR for cross-presentation of IgG IC, FcγR were found to be critical for FcRn-mediated cross-presentation by enabling the initial cell surface binding of IgG IC and facilitating internalization of the complexes into FcRn-containing endosomes or phagosomes. The physiological relevance of this process was established by subsequent *in vivo* studies demonstrating the importance of FcRn in establishing system-wide immunity resulting from cross-priming and conferring protection from colorectal cancer (CRC) (112, 113). These data provided conclusive evidence that FcRn enables cross-presentation of IgG-complexed antigens downstream of FcγR and that this is a physiologically meaningful process, which is active in initiating immune responses to low doses of antigen in the early stages of infection.

The differential ability of FcRn to enable cross-presentation in CD8^−^CD11b^+^ versus CD8^+^CD11b^−^ DC was found to reflect the known biology of these two DC subsets. Given that the IgG–FcRn interaction requires an acidic pH, the neutral pH that is present in endocytic compartments of CD8^+^CD11b^−^ DC is not conducive to strong ligand receptor interaction. In contrast, the intermediate endosomal acidity level of CD8^−^CD11b^+^ DC between that of macrophages and CD8^+^CD11b^−^ DC provides an ideal binding environment for FcRn binding to IgG IC (6, 9). The importance of acidification to FcRn-mediated cross-presentation was shown by its inhibition in the presence of vacuolar ATPase (V-ATPase) inhibitors and by the active enrichment of V-ATPase on the phagosomal membranes of compartments in which FcRn was crosslinked by IgG IC but not on those containing IHH-IgG IC (60). Nonetheless, the process by which FcRn enables cross-presentation employs much of the same molecular machinery as does the cross-presentation of soluble antigen by CD8^+^ DC. Specifically, FcRn ligation by IgG IC led to the selective enrichment of the NOX2 component gp91-phox, the Sec61 retrotranslocator, the TAP transporter, and the Rab27a GTPase to phagosomal membranes. Furthermore, FcRn-mediated cross-presentation required cytosolic export of antigen and proteasomal processing. In addition to directing IgG IC toward specialized antigen processing mechanisms, which lead to the surface presentation of epitope-loaded MHC-I, cross-linking of FcRn by IgG IC induced a signaling cascade, resulting in enhanced secretion of the cytotoxicity promoting cytokine IL-12, thereby providing an additional stimulus to encourage activation of antigen-primed and specific CD8^+^ T cells (113). FcRn within CD8^−^CD11b^+^ DC thereby enables cross-presentation of antigen within IgG IC via multiple mechanisms. Among others, these include trafficking of complexed antigen into a processing pathway that promotes epitope conservation but which is not the default pathway for soluble antigens in CD8^−^ DC.

Identification of FcRn as a necessary facilitator for IgG IC cross-presentation provides an important intracellular mechanistic explanation for numerous demonstrations of the ability of IgG IC to activate CD8^+^ T cells. Similarly, existing studies not having directly examined FcRn but having looked at IgG IC-mediated cross-presentation can provide valuable information about additional processes that are likely to be involved in FcRn-mediated cross-presentation. The existence of an antigen storage compartment that persists for a prolonged period of time within IgG IC-loaded CD8^−^ DC is not only consistent with the slower phagosome-to-cytosol release kinetics observed for FcRn-mediated antigen presentation but also suggests that the stable binding of FcRn to IgG IC in the acidic endolysosomal environment might contribute to creating a durable reservoir of immunostimulatory antigen (60, 114). With respect to additional DC subsets, pDC, which cross-present soluble antigen poorly, display significantly enhanced cross-presentation when antigens are targeted to the surface FcγR in the form of IgG IC (22, 115). Given the dependence of IgG IC cross-presentation on intracellular processing downstream of FcγR-mediated uptake, this finding predicts a role for FcRn in the cross-presentation of IgG IC by pDC. Furthermore, studies on human DC subsets have demonstrated enhanced cross-presentation when antigen is delivered in the form of an IgG complex, thus, indicating that FcRn’s ability to enhance CD8^+^ T cell activation via cross-priming extends to human DC (22, 116). Direct proof of FcRn’s ability to enhance MHC-I driven activation of CD8^+^ T cells and MHC-II driven activation of CD4^+^ T cells in humans will have significant therapeutic implications based on the number of physiological processes shown experimentally to be regulated by FcRn-mediated antigen presentation.

### Physiological Significance of FcRn-Mediated Antigen Presentation

Fc receptor-mediated antigen presentation plays a significant role in the regulation of physiological processes that contribute to health and disease. Indeed, an important positive feedback loop exists between FcRn-mediated MHC-II antigen presentation and the production of IgG by B cells. Targeting antigen to DC is a known mechanism to induce CD4^+^ T cell help, which is essential for antibody responses (117). This process of driving T cell help for promoting humoral immunity has been shown to be further accelerated when antigen is delivered to DC in the form of an IgG IC, which stimulates FcγR-mediated antigen uptake (118). That this process is dependent on FcRn-mediated processing of the IgG IC itself has been shown in a series of studies using an animal model in which the bovine FcRn (bFcRn) has been overexpressed in transgenic mice (119, 120). Immunization of bFcRn transgenic mice, even using weakly immunogenic antigens, led to significantly greater expansion of antigen-specific B cells and plasma cells compared to immunization of WT mice, a finding that is consistent with the binding of bFcRn to mouse IgG (119–122). Importantly, not only was production of antigen-targeted antibody increased in bFcRn overexpressing mice but significantly greater diversity in the antibody repertoire was seen following immunization (119). Given that increased antigen-specific antibody production can be expected to lead to greater amounts of antigen presentation by FcRn and thus feed back to even greater antibody synthesis, these results demonstrate that FcRn contributes to shaping the production and diversity of its own ligand and thus serves an important role in both cellular and humoral immunity.

The end result of FcRn-mediated antigen presentation is often a powerful pro-inflammatory response which can be either helpful or harmful depending on the context. Opsonization of infectious agents is a critical parameter in the eradication of many pathogens and, while protection is partially attributable to antibody dependent cellular cytotoxicity (ADCC), evidence indicates that FcRn-mediated antigen presentation also plays a significant role. FcRn has been directly implicated in protection from intestinal *C. rodentium* infection in a model in which CD4^+^ T cell activation and IgG are critical parameters in successful pathogen clearance (97). Additionally, FcRn contributes to CD4^+^ and CD8^+^ T cell activation in response to vaccination with IgG opsonized and inactivated *Francisella tularensis* (123, 124). Upon rechallenge with *F. tularensis*, mice having been vaccinated with the opsonized microbes were significantly more protected from this intracellular pathogen than microbe alone controls. FcRn-mediated protection from infection in response to IgG-complexed microbial antigens is not only limited to bacterial pathogens but also extends to viral infections such as influenza, herpes simplex, and HIV model viruses (125–127). Importantly, FcRn-mediated protection induced by vaccination against these viruses was shown to result in the generation of long-term memory T cell responses, thereby confirming a mechanistic role for antigen presentation in the process (126). Critically, this body of work has firmly established that T cell-mediated protection against microbial and viral antigens that is initiated by IgG-complexed antigen, depends upon the antigen presentation function of FcRn within DC. Thus, FcRn-mediated antigen presentation provides an intracellular mechanism that explains the findings of many other studies documenting IgG IC-driven protection from infection. Among other pathogens, targeting antigen in the form of IgG IC toward FcγR has been shown to be effective against human papilloma virus (HPV) and *Salmonella typhimurium* via a mechanism which is most likely also dependent upon FcRn following initial IgG IC uptake (128–131). The antigen presentation function of FcRn in the context of invasive pathogens is thus highly beneficial to the host, particularly since its high sensitivity enables early immunological activation upon infection.

The exquisite sensitivity of FcRn to enable the presentation of small doses of antigen renders it a highly potent immunostimulatory molecule. While this is advantageous in the context of a foreign pathogen, FcRn-mediated antigen presentation can serve to promote pathological inflammation in the context of autoimmune or allergic diseases. Antibody-enhanced cross-presentation of self-antigens expressed by pancreatic β cells can drive the development of CD8^+^ T cell-mediated autoimmune diabetes by breaking tolerance to endogenous molecules (132). Similarly, antigen uptake by FcγR in DC has been shown to contribute to allergic airway hyper-responsiveness and inflammation by driving CD4^+^ T cell activation via a mechanism that almost certainly involves FcRn (133). Indeed, two additional studies have demonstrated a direct effect for FcRn in the regulation of allergic airway inflammation although they did not identify the cell compartment in which FcRn was acting (134, 135). Perhaps the most conclusive evidence for the ability of FcRn-mediated antigen presentation to promote inflammation comes from studies conducted in the intestine where FcRn has been shown to drive colitis in the presence of anti-microbial IgG against the common microbial flagellin protein (136). Whereas WT mice with high titers of anti-flagellin IgG due to immunization with this ligand developed severe colitis upon intestinal epithelial barrier breakage by dextran sodium sulfate (DSS), *Fcgrt*^−/−^ mice were largely protected from severe weight loss and tissue damage in this model. Reciprocal bone marrow chimeras in which serum IgG levels were normalized across all experimental groups not only demonstrated that protection from intestinal damage was not due to lower anti-flagellin IgG titers in *Fcgrt*^−/−^ animals but also identified the hematopoietic compartment as the source of FcRn-mediated inflammation. Further study revealed that CD8^−^CD11b^+^ DC from the intestines of WT colitic mice induced far greater CD8^+^ T cell activation *in vitro* and *in vivo* compared to DC from *Fcgrt*^−/−^ mice, directly indicating the importance of FcRn in driving pro-inflammatory T cell responses (60). In spite of the extensive tissue damage that results from FcRn-mediated intestinal inflammation, however, WT mice are significantly protected from the development of inflammation-associated CRC in comparison to their *Fcgrt*^−/−^ littermates in whom the protection from FcRn-mediated inflammation translated into dampened anti-cancer immunity (113). Consistent with the widely recognized importance of CD8^+^ T cells in conferring anti-tumor immunity, depletion studies in conjunction with a DC-specific deletion of FcRn conclusively identified FcRn-mediated cross-presentation as the mechanism by which tumor protection was enabled (98, 113). While numerous studies had previously shown that vaccination strategies utilizing IgG-complexed tumor antigens were far superior in driving tumor-protective immunity compared to soluble tumor antigen, this was the first demonstration that FcRn in DC is an active and important part of the endogenous anti-tumor immunosurveillance system (137).

## Lingering Questions in FcRn-Mediated Antigen Presentation

An important physiological role for FcRn-mediated antigen presentation has been conclusively demonstrated and many of the key intracellular processes involved have been identified. Nonetheless, many valuable questions remain unanswered. How is FcRn-mediated antigen presentation affected by additional stimuli such as temporally or spatially proximal TLR-ligation within the IgG IC-containing DC or the presence of mixed immune complexes containing immunoglobulins of different subclasses of IgG or immunoglobulin isotypes (138, 139)? How much impact do characteristics of the IgG IC, such as ligand composition, IgG-to-antigen ratios, and IgG IC size, have upon the immunological outcome of FcRn-mediated antigen presentation (140–143)? Are FcRn-trafficked IgG-complexed antigen processed within the same intracellular compartment for MHC-I and MHC-II loading or are IgG IC routed to distinct endocytic compartments for each of these processes (106, 144–146)? How is FcRn trafficked in different subsets or types of APCs and how does this relate to the aforementioned questions? Addressing these and other queries has important implications for the development of FcRn-targeted therapeutics that could theoretically be tailored to trigger CD4^+^ T cells for an extracellular bacterial infection or CD8^+^ T cells for tumor eradication. Our increasing understanding of the structural details of the FcRn–IgG interaction open significant possibilities for therapeutic development (80, 87) that can best be applied and exploited by developing a complete understanding of the mechanisms that govern FcRn-mediated antigen presentation.

## Conflict of Interest Statement

The authors declare that the research was conducted in the absence of any commercial or financial relationships that could be construed as a potential conflict of interest.
